# Everybody loves sugar: first report of plant feeding in triatomines

**DOI:** 10.1186/s13071-016-1401-0

**Published:** 2016-02-29

**Authors:** Hector Manuel Díaz-Albiter, Tainá Neves Ferreira, Samara Graciane Costa, Gustavo Bueno Rivas, Marcia Gumiel, Danilo Rufino Cavalcante, Márcio Galvão Pavan, Marcelo Salabert Gonzalez, Cícero Brasileiro de Mello, Viv Maureen Dillon, Rafaela Vieira Bruno, Eloi de Souza Garcia, Marli Maria Lima, Daniele Pereira de Castro, Rod James Dillon, Patricia de Azambuja, Fernando Ariel Genta

**Affiliations:** Laboratório de Bioquímica e Fisiologia de Insetos, Instituto Oswaldo Cruz, FIOCRUZ, Rio de Janeiro, Brazil; Laboratório de Biologia Molecular de Insetos, Instituto Oswaldo Cruz, FIOCRUZ, Rio de Janeiro, Brazil; Departamento de Biologia Geral, Universidade Federal Fluminense, Niterói, Brazil; Laboratório de Epidemiologia e Sistemática Molecular, Instituto Oswaldo Cruz, FIOCRUZ, Rio de Janeiro, Brazil; Instituto Nacional de Ciência e Tecnologia em Entomologia Molecular, Rio de Janeiro, Brazil; Institute of Integrative Biology, Biosciences Building, University of Liverpool, Crown Street, Liverpool, L69 7ZB UK; Laboratório de Ecoepidemiologia da Doença de Chagas, Instituto Oswaldo Cruz, Rio de Janeiro, Brazil; Division of Biomedical and Life Sciences, Faculty of Health and Medicine, Lancaster University, Lancaster, UK

**Keywords:** *Rhodnius prolixus*, Chagas disease, *Trypanosoma cruzi*, Triatomine, Phytophagy, Sugar meal

## Abstract

**Background:**

Triatomines, which are the vectors of *Trypanosoma cruzi*, have been considered to be exclusive blood feeders for more than 100 years, since the discovery of Chagas disease.

**Methods:**

We offered artificial sugar meals to the laboratory model-insect *Rhodnius prolixus*, which is considered a strict haematophagous insect. We registered feeding by adding colorant to sugar meals. To assess putative phytophagy, fruits of the tomato *Solanum lycopersicum* were offered to *R. prolixus* and the presence of tomato DNA was assessed in the insects using PCR. We also assessed longevity, blood feeding and urine production of fruit-exposed triatomines and control insects.

**Results:**

All instars of *R. prolixus* ingested sugar from artificial sugar meals in laboratory conditions. First instar *R. prolixus* ingested plant tissue from *S. lycopersicum* fruits, and this increased the amount of blood ingested and urine excreted. Decreased mortality was also observed after blood feeding. Exposure to *S. lycopersicum* increased longevity and reduced weight loss caused by desiccation.

**Conclusions:**

We describe here the first report of sugar feeding and phytophagy in a species that was considered to be a strict blood-feeder for over a century. We suggest that local plants might be not merely shelters for insects and vertebrate hosts as previously described, but may have a nutritional role for the maintenance of the triatomine vectors. The description of sugar and plant meals in triatomines opens new perspectives for the study and control of Chagas Disease.

## Background

American Trypanosomiasis (Chagas Disease) is a neglected illness affecting 8 million people mainly in the Americas, but its geographic spread by human migration has gained attention [1]. It is a chronic and severe disease caused by the haemoflagellate *Trypanosoma cruzi* and there is no vaccine for it [1]. Since its discovery, American Trypanosomiasis has been clearly incriminated as a vector-borne disease [2] transmitted by hematophagous insects from the family Triatominae. Vectorial transmission of *T. cruzi* occurs when metacyclic-infected triatomine faeces contacts abraded skin in the bite area, oral or nasal mucosae as well as conjunctiva of the mammalian host [1].

The development of vector control strategies has resulted in a dramatic reduction in new vector-borne cases in endemic countries [3]. However, in the past decade there have been a series of re-infestation events of insecticide-treated houses by sylvatic triatomine populations in Bolivia, Colombia and Venezuela. There have also been outbreaks in different areas of Brazil, Colombia and Venezuela attributed to oral infection associated with consumption of contaminated food, particularly of plant origin, such as sugar cane and fruits such as guava and açai [4–8].

The Triatominae is a numerous and diverse group that has colonized temperate, subtropical and tropical ecotopes, mainly in the Americas [9]. This group contains several species that have adapted to an array of natural habitats and includes some genera that show strong association with particular vegetation. For example, *Rhodnius* spp. are highly associated with palm trees and bromeliads [10–12]. Triatomines display a highly diverse array of fluid-sucking dietary habits. *Triatoma rubrofasciata* actively feeds on rodents in northeast Brazil, whereas in southeast Asia, it feeds preferentially on caterpillars [13]. Other triatomines are able to complete their life-cycle feeding on either mammal or invertebrate blood [14]. *Eratyrus mucronatus* is able to feed on arachnids and mammals. Some species even display cleptohaematophagia, feeding on other blood-engorged triatomines [12, 14, 15].

It is widely accepted that predatory reduviids evolved from phytophagous species about 230 million years ago and that haematophagy emerged in triatomines about 85 mya as an opportunistic specialization of predatorial reduviids, probably shifting from sucking fluids from other insects, to probing vertebrate blood and then becoming fully haematophagous [16]. Interestingly, there are records of facultative haematophagy in otherwise phytophagous hemipterans, a phenomenon that has not been fully explored [17, 18].

Triatomines were key models in the early investigations of insect physiology, especially *Rhodnius prolixus*, which was used by Sir Vincent Wigglesworth in studies on the neuroendocrinal control of moulting [19]. *R. prolixus* is an important model for triatomine biology, including studies on parasite-vector interactions, biochemistry, physiology, behaviour and microbiology, culminating in the sequencing of its genome [20]. Triatomines are considered strictly hematophagous, as in normal conditions an engorging blood meal is enough and necessary for larval development, moulting and female oviposition. In this respect they differ from other insect vectors such as mosquitoes or phlebotomine sand flies, whose adults consume both plant sugar and blood meals [20].

The association of triatomines with plants was always considered a secondary effect derived from the habitat preference of their vertebrate hosts. Field-collected triatomines are usually found in a very poor nutritional status, withstanding long periods of fasting, even when they have been collected from microhabitats colonized abundantly by small mammals [10].

In this report, we investigated if phytophagy could have an impact on the fitness of *R. prolixus*. As a proof of concept, we tested if *R. prolixus* was able to ingest artificial sugar meals and recovered plant DNA from the gut of *R. prolixus* fed with cherry tomatoes (*Solanum lycopersicum*). We chose *S. lycopersicum* as it is an easy fruit to obtain throughout the year without pesticide or insecticide contamination, being included in the Brazilian organic certification programs. Also, *S. lycopersicum* has available genetic information enabling us to design probes for PCR detection of ingested plant tissue. Exposure of *R. prolixus* to a plant water and nutrient source had significant effects on physiological parameters, such as lifespan, weight, blood meal size, survival and urine production.

## Methods

### Artificial sugar meals

Twenty insects of each instar (first, second, third, fourth, fifth instar nymphs and male or female adults) were kept separately in a 100 mL glass container and offered a piece of cotton (0.2 g) wetted with 2 mL of a 10 % (w/v) sucrose solution containing 0.5 % (w/v) bromophenol blue. Control bugs were exposed to a 0.2 g cotton wetted with 2 mL of 0.5 % (w/v) bromophenol blue solution in water. After 3 days at room temperature and humidity, insects were withdrawn, anesthetized in ice and dissected with cold 0.9 % (w/v) NaCl under a stereomicroscope. Guts were pulled apart, washed with saline and photographed using a Luxeo 4Z stereomicroscope equipped with the Software Labomed Pixel Pro.

### Tomato feeding DNA extractions

One hundred insectary reared, newly emerged first instar *Rhodnius prolixus* were transferred into 100 mL glass containers with folded filter paper. Insects underwent a fasting period of 15 days. After that, one cherry tomato, produced in insecticide-free conditions, was placed into the glass container and insects were allowed to feed *ad libitum*. Ten insects were randomly collected every day for five consecutive days and washed twice in distilled water to eliminate possible foreign DNA contamination. Insects were placed in 1.5 mL Eppendorf vials, flash frozen in liquid N_2_ and kept at −20 °C for further use. Individuals from a separate container with no tomatoes were used as negative controls. DNA extraction was carried using the QIAmp (QIAGEN) DNA extraction kit following the manufactures protocol for total DNA extraction from tissues. Briefly, 10 first instar *R. prolixus* were homogenized in 100 μL of PBS to which 100 μL of buffer ATL were added and mixed for 10 s. 20 μL of proteinase K were then added and samples were then incubated at 56 °C for 3 h, vortexing every 30 min. 200 μL of AL buffer were then added and the samples were vortexed for 15 s and centrifuged at maximum speed for 1 min to get rid of debris. The supernatant was then recovered and applied to a kit column by centrifuging at 6000xg. Samples were then washed with 500 μL of buffer AW1 and AW2 and DNA was recovered by elution with 200 μL of nuclease-free water. Tomato specific primers Le1 and Le2 (sequences 5′-CCGAGGCGCGCAAGCTCTTC-3′ and 5′-TAAAGCCTTGCGGCGTGCGAG-3′, respectively) were used to amplify a 332 base-pair (bp) fragment of the *S. lycopersicum* first and second internal transcribed spacer (ITS-1 and ITS-2) regions. Components of the amplification reactions were placed in PCR buffer with 0.1 mM dNTP, 5 μM of each primer and 1.0 U of Go-Taq DNA polymerase (Promega) in a total volume of 25 μL. Touch-down PCR conditions were as follows: initial denaturation at 94 °C for 3 min followed by 20 cycles of 94 °C for 30 s, 70 °C for 1 min with decrements of 0.5 °C every cycle until 60 °C and 72 °C for 1 min. This was followed by another 20 cycles of 94 °C for 30 s, 60 °C for 30 s and 72 °C for 1 min and a final extension step of 72 °C for 5 min. Samples were resolved on 1.5 % (w/v) agarose stained with ethidium bromide and viewed with an ultra-violet light transilluminator. As a positive control, DNA was extracted directly from tomatoes as described in ref. [21].

To rule out the possibility of external DNA contamination after rinsing twice with distilled water, 100 newly emerged first instar *Rhodnius prolixus* were transferred into 100 mL glass containers with folded filter paper. Insects underwent a fasting period of 15 days. After that, they were anesthetized on an ice-cold glass petri dish cover and their proboscides tip (half of the first segment) were severed using a scalpel. Insects were then transferred to a 100 ml glass container and offered one cherry tomato, produced in insecticide-free conditions, as above. Ten insects were randomly collected after 24 h and washed twice in distilled water to eliminate possible foreign DNA contamination. Insects were placed in 1.5 mL Eppendorf vials, flash frozen in liquid N2 and kept at −20 °C for further use. Regular individuals from a separate container with tomato were used as a control. Total DNA was extracted and tomato-specific sequences were amplified as stated above.

### Insect survival during tomato exposure

For each replicate in all assays we used 60 laboratory-reared first instar *R. prolixus* that were pooled in three groups of 20 individuals each and transferred into three separate 100 mL glass containers with folded filter paper as a substrate. One small (approximately 2 cm in diameter) tomato (*S. lycopersum*) grown under insecticide-free conditions was placed inside each pot. Tomatoes were washed twice with distilled water prior to the experiments to remove any possible contamination. Tomatoes were changed every week or until signs of rotting were noticeable. Control groups were set in the same conditions of experimental insects but without tomatoes. Other groups were assembled with a hanging (inaccessible) tomato inside the pot, and the contact of insects with the tomato was impeded by wraping the fruit with cloth. All experiments were performed in an insectary room with regulated temperature (28 °C) and relative humidity (60–70 %). The mortality was registered daily in all conditions until death of all individuals. Data collected from the experiments is representative of three different biological replicates.

### Changes in insect weight and blood-feeding after tomato exposure

Thirty-day first-instar survivor nymphs had their weights registered. To find out whether previous tomato-exposure might affect blood-feeding, a defibrinated blood-meal was offered through a latex membrane attached to a glass case heated at 37 °C [22] for survivor specimens of *R. prolixus* after 30 days of tomato exposure. Insects were weighed 2 h post blood-feeding (PBF). Insect mortality was registered 24 h PBF as well as the weight of survivors. Urine production was estimated by subtracting insect’s weight 24 h PBF from weight 2 h PBF. Data from experiments represented two different biological replicates.

### Statistical analysis

Survival analyses were performed using the Log-rank (Mantel-Cox) Test. Comparison between means of two independent groups was done with an unpaired Student *t*-test. One-way ANOVA followed by Bonferroni post-hoc comparison tests were performed to compare three independent groups. Proportions were compared using Fisher’s exact test. For nonparametric data, pairwise comparisons were analyzed with a Mann-Whitney test. Results are expressed as group mean ± SEM. Significance was considered at a level of *p* < 0.05. All data were analysed using GraphPad Prism software (version 5.0, GraphPad Software Inc.).

### Ethics

All procedures involving the maintenance of triatomines and experiments using rabbit blood were approved by Ethics Committee in Animal Experimentation (CEUA/FIOCRUZ) under the protocol number L-0061/08.

## Results

### *Rhodnius prolixus* actively ingests artificial sugar meals

**Fig. 1:**
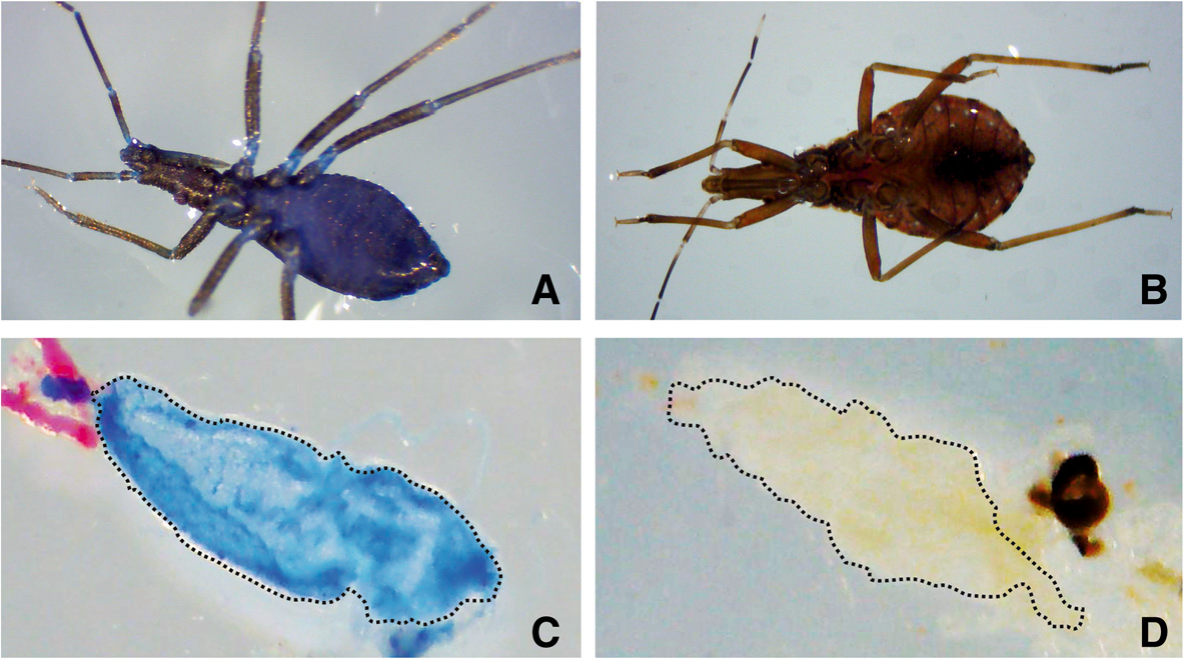
Ingestion of artificial sugar meals by 1st instar nymphs of *Rhodnius prolixus*. Animals were exposed to cotton wool soaked with bromophenol blue solution (1 % w/v) plus sucrose 10 % (w/v) (**a** and **c**, experimental) or bromophenol blue solution (1 % in water) (**b** and **d**, controls). Photos are representative insects or guts from groups of 20 insects. The experiment was repeated three times. **a** and **b**, whole insects. **c** and **d**, dissected guts. The dotted line shows the limits of the anterior midgut

In laboratory conditions, plant sugar meals were mimicked using a piece of cotton wool wetted with a sucrose solution. This is currently done for mosquitoes, sand flies and other hematophagous dipterans [23, 24]. This method was used to check if triatomines could take artificial sugar meals. We recorded *R. prolixus* ingestion of sugar solution by adding a colorant (bromophenol blue) to the meals. First instar nymphs exposed to sugar meals became visibly blue (Fig. 1a), suggesting that the colorant was ingested and diffused to the hemolymph. Control animals exposed to the same conditions, but with cotton wool wetted only with colorant did not show any sign of staining within the body (Fig. 1b), which excludes the possibility of accidental staining of the external cuticle. To confirm that the colorant was acquired by ingestion of sugar solutions, bugs were dissected and washed with saline, revealing an intense blue coloration inside the gut of the experimental insects (Fig. 1b), in comparison there were no changes in control colouration (Fig. 1d). The same results were obtained with second, third, fourth and fifth instar nymphs, and also with male and female adults (data not shown).

### *Rhodnius prolixus* actively ingests plant tissue under laboratory conditions

**Fig. 2:**
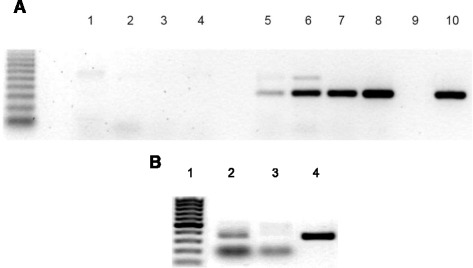
DNA analysis of 1st instar *R. prolixus* nymphs exposed to tomato *ad libitum*. **a** Image shows negative controls (1–4) and insects (5–8) collected after 24, 48, 72 and 96 h respectively of exposure to tomatoes. Lane 9 is a PCR negative control and lane 10 is a positive control using tomato-extracted DNA. **b** DNA analysis of 1st instar *R. prolixus* nymphs with cut proboscis tip exposed to tomato *ad libitum*. Image shows regular 1st instar *R. prolixus* nymphs (lane 2) and insects which proboscis was cut before exposure to tomato (lane 3). All insects were collected after 24 h of exposure. Lane 4 is a PCR positive control using tomato DNA and lane 1 is DNA molecular weight marker (100 bp ladder)

To test if *R. prolixus* would be able to ingest tissue of plant origin, we designed an experiment with first instar insects and so-called ‘cherry’ tomatoes (*S. lycopersicum*). After 1 day of incubation with cherry tomatoes, we detected tomato DNA inside first instar nymphs of *R. prolixus* (Fig. 2a lanes 5–8), with no evidence of any background, non-specific amplification in control bugs (Fig. 2a lanes 1–4). Additionally, we were not able to detect any tomato DNA from insects with the proboscis tip removed, after exposure to tomatoes in the same conditions (Fig. 2b), suggesting that the amplified tomato DNA was ingested by the triatomines.

### Phytophagy affects aspects of *R. prolixus* physiology

After detection of *S. lycopersicum* DNA in *R. prolixus*, we decided to investigate if phytophagy had any impact on physiological parameters of this insect. We evaluated the lifespan of first instar *R. prolixus* nymphs exposed to *S. lycopersicum* fruits. To investigate whether positive physiological effects on insects were caused by actual tomato ingestion and not by fruit-derived humidity, we examined the lifespan of insects that were a) allowed direct contact with the fruit (accessible tomato group), b) exposed to tomato but denied direct contact with the fruit by hanging it inside the pot (inaccessible tomato group), and c) had no tomato at all (no tomato group).

**Fig. 3:**
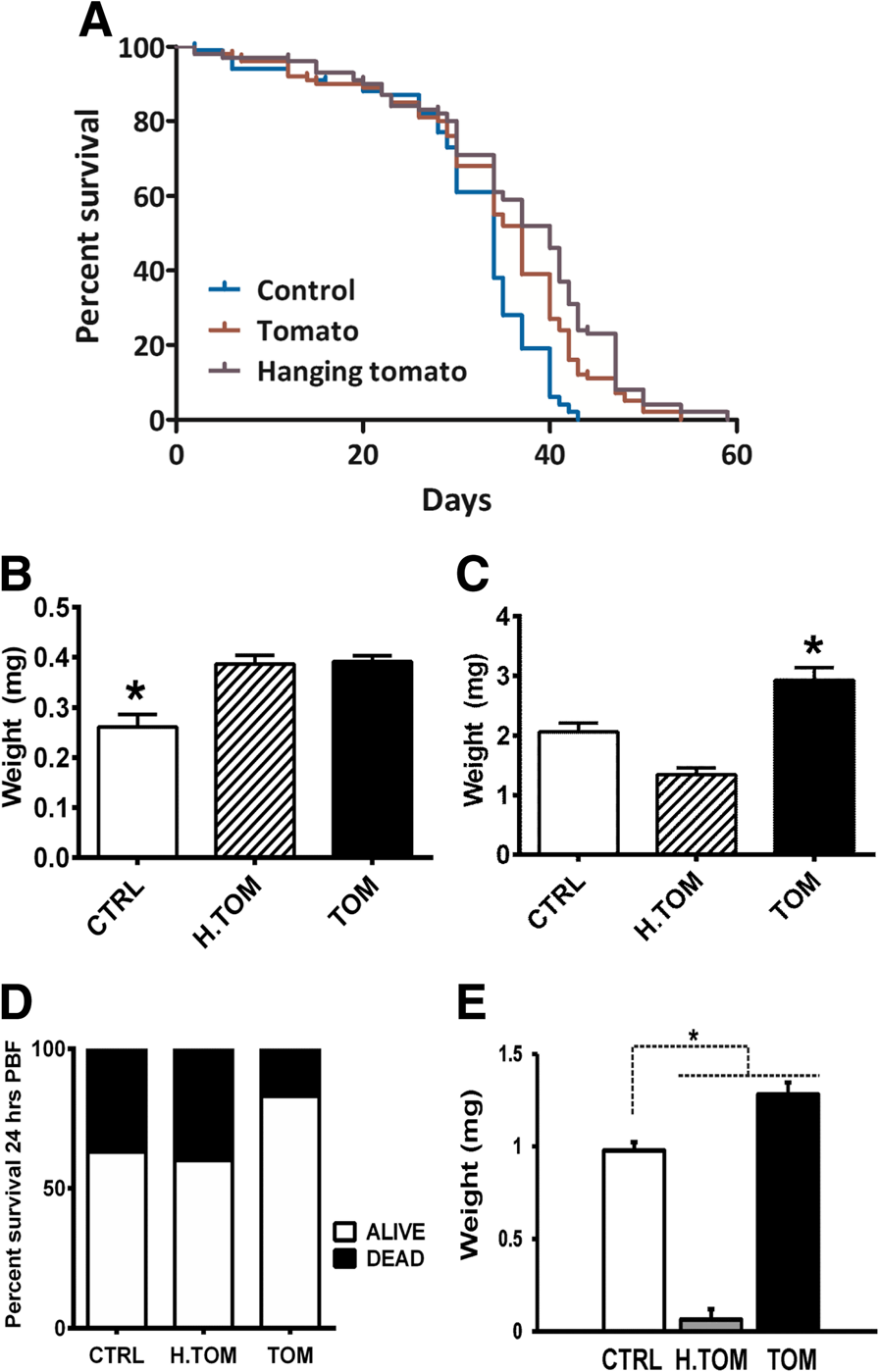
The effect of exposure to plant nutrient sources on physiological parameters of *Rhodnius prolixus*. **a** Survival curves for 1st instar colony-reared *R. prolixus* exposed to tomato fruit, hanging tomato (inaccesible to bugs) or no food source (Control). Survival was recorded until death of all individuals. Survival curves represent data from 5 different biological replicates each with 20 individuals (total *n* = 300). Average life span values (±SEM) in controls, hanging tomato and tomato groups were 31.2 ± 0.9, 36 ± 1 and 34 ± 1 days, respectively. Average life spans in each replica ranged from 28.3 to 34.5 days (control), 30.9 to 43.15 days (hanging tomato) and 31.4 to 35.9 days (tomato). **b** Insect weight after 30 days (total *n* = 140). **c** Weight 2 h Post Blood Feeding (PBF) (total *n* = 130). **d** Mortality 24 h PBF (total *n* = 130). **e** Urine produced 24 h PBF (total *n* = 110). CTR – Controls with no food source. TOM – tomato. H. TOM – hanging tomato. Asterisk denotes groups which are significantly different from the other conditions tested (*p* < 0.05). Error bars in (**b**), (**c**) and (**e**) are SEM

As observed in Fig. 3a and b, the presence of a fruit inside experimental pots had an impact in lifespan and weight of *R. prolixus*. Mean lifespan of no tomato groups was shorter (31.29 ± 0.92 days) compared with accessible and inaccessible tomato groups (34.17 ± 1.09 and 36.47 ± 1.14 days, respectively). Survival (*p* < 0.05, log-rank test) and weight (*p* = 0.001, ANOVA, 30 days post-tomato exposure) of no tomato *vs* accessible tomato groups was significantly lower. These effects could be attributed to an increase in micro-environmental humidity, since allowing contact of insects with the fruit did not increase lifespan of inaccessible *vs* accessible tomato groups (*p* > 0.05, log-rank test). However, only insects that were allowed *ad libitum* access to the fruit exhibited a significant increase in weight (accessible tomato = 2.93 mg, no tomato = 2.07 mg, inaccessible tomato = 1.35 mg) after blood feeding (Fig. 3c, *p* < 0.05, ANOVA), lower mortality after blood feeding (Fig. 3d, *p* = 0.0007, Chi-square), as well as higher urine production (Fig. 3e, *p* < 0.05, unpaired *t*-test). Surprisingly, insects exposed to the hanging tomato produced a smaller amount of urine (Fig. 3e, *p* < 0.05, unpaired *t*-test), but this could be related to the smaller amount of blood ingested (Fig. 3c, *p* < 0.05, ANOVA). It is highly unlikely that the positive effects observed in the group with access to the tomato were due to humidity from fruits inside the experimental pots, as shown by data from the inaccessible tomato groups. These results strongly suggest that the overall healthier physiological status in accessible tomato *R. prolixus* is explained by phytophagy.

## Discussion

Artificial sugar meals are a common resource used for maintenance of mosquitoes and other hematophagous dipterans in the laboratory. They were never considered as necessary for triatomine colonies due to the high resistance they have to starvation and dehydration and the belief that these bugs are obligatory hematophages. However, our observation that *R. prolixus*, a model triatomine, is able to take artificial sugar meals, opens potential strategies for better colony maintenance of triatomines, which are difficult to raise under artificial conditions, and it creates new possibilities for testing the interference of soluble compounds in triatomine physiology and the development of *T. cruzi* inside its vector. Interestingly, insects which have access to water under the same conditions did not show any sign of ingestion, suggesting that *R. prolixus* is not drinking the solution in the cotton wool just for acquisition of water. Our results suggest the presence of a previously unrecorded behavioural trait, a specific detection for sugar molecules in food in triatomines that deserves more detailed investigation in the future.

In nature, the opportunity for triatomine bugs to feed on an alternative food source such as plants, would provide a hitherto unexpected nutritional benefit when compared to bugs exclusively reliant on animal food sources. Our results, showing improvement in physiological parameters of triatomines after putative phytophagy could be related to poor nutritional status in starving insects, and partial reversion of this condition by occasional feeding on plants. Autophagy of body tissues, mainly fat body, salivary glands and gut normally occurs in starved insects [25]. Starved insects are less likely to escape predation, mate or reach food sources [26, 27]. Insects that have access to additional source of energy would possess a physiological advantage over starving insects. A previous study showed that artificial supplementation with sucrose stimulated the obligate blood feeding *Simulium venustum* and had a positive effect on engorgement leading to higher volume of blood consumed in haematophagous bugs and this led to a positive effect on survival [28]. Similarly, previous sugar feeding increases the longevity and fecundity of *Phlebotomus papatasi* females after a blood meal [29]. Our results suggest that even a small amount of nutrients or water present in plants could improve the fitness of triatomines which putatively feed on these food sources. These insects might remain healthier for longer periods of time and feed on blood more successfully. A longer life will also mean that they are more likely to bite a second host and therefore more likely to successfully transmit disease. It should be stressed that according to our data, plant nutrient sources are not sufficient for triatomines to undergo full development. We believe that plant nutrients are complementary and only extend the insect survival during fasting periods between blood meals.

Phytophagy could only be a metabolic improvement if *R. prolixus* had the proper enzymatic arsenal to successfully absorb plant carbohydrates. A recent *R. prolixus* gut transcriptome analysis described several highly expressed (>10-fold) carbohydrases which were thought to be associated with bacterial cell wall digestion [29]. Intriguingly, an abundant number of transcripts for alpha-amylase, an enzyme specific for plant polysaccharide hydrolysis, were found [30]. Additionaly, it has been found that a *R. prolixus*α-glucosidase plays a role in hemozoin formation [31]. The role of Triatominae gut α-glucosidase in the digestion of plant sugars deserves more detailed investigations, and could be an evolutionary conserved trait from hemipterans phytophagous ancestors. A similar rationale could be applied to the use of the sucking buccal apparatus to the perforation of the fruit pericarp, as could be inferred from our experiments with *S. lycopersicum*.

Plant DNA was recovered from first instar nymphs of *R. prolixus* which were exposed to fruits of *S. lycopersicum.* This plant nutrient source (cherry tomatoes) was used in this proof-of-concept experiment because it was one of the few insecticide-free fruits commercially available throughout the year in Brazil. Our experiments showed specific amplification of tomato DNA from whole bugs which had *S. lycopersicum ad libitum* as a putative nutrient source. Despite the fact that our results showed that it was highly probable that tomato DNA was ingested by the bugs, it was impossible to disregard the possibility that tomato DNA was adhered to insect surface. Dissections and amplification of DNA from isolated guts could have solved this issue. However, dissections of first instar nymphs hardly result in non-contaminated tissues. First instar nymphs are very fragile and manipulation of their body result in exposition of dissected guts to saline which was used to wash the surface of the animal, or other body fluids.

To rule out external surface contamination with tomato DNA, and amplification of non-ingested material, we performed an experiment with bugs which had the tip of proboscis surgically removed. Those insects, after *ad libitum* exposure to *S. lycopersicum* fruits, did not show any tomato DNA, as exhibited by negative PCR amplification. This result strongly suggests that the tomato DNA amplified from first instar nymphs was acquired by ingestion. This evidence indicates that, besides being capable of ingesting artificial sugar meals, triatomines can acquire nutrients by perforation of plant tissues. In this context, fruits might be considered a putative natural alternative source of additional nutrients for triatomines. Interestingly, many tropical fruits present soft pericarps. This is the case of palm tree fruits like açaí, which is a common source of *Trypanosoma cruzi* contaminated food in the north of Brazil. The association of triatomines with palms has been well documented [10, 12]. Palm leaves are used for roof covering of local dwellings, and the fruits are used for human consumption. Our results suggest that acquisition of nutrients from flowers or fruits might be an additional explanation to the association of triatomines with tropical plants.

Physiological midgut conditions in triatomines and their changes by starvation or blood feeding affect the differentiation of *T. cruzi* from epimastigotes into metacyclic trypomastigotes. A long starvation period causes population reduction in the rectum, without total elimination and an increase in the percentage of metacyclic trypomastigotes [32, 33]. Blood feeding initially reduces the density of metacyclic tripomastigotes [34]. If this hypothetical phytophagy occurs in the Triatominae, studies addressing the effect of plant tissue ingestion in gut dwelling *T. cruzi* should be performed, Specifically, experiments analysing how plant tissue ingestion by triatomines that have experienced long starvation periods might affect *T. cruzi* populations and their infectivity to mammals.

In recent reports, the association of triatomines with obligatory symbionts has been questioned by the observation of a complex gut microbiota in field insects [35]. It is possible that the association of laboratory-reared *R. prolixus* with *Rhodococcus rhodni*, a gut-dwelling actinomycete which supplements the insect vitamin B metabolism [36], is related to the absence of alternative sources of nutrients. The association of *R. prolixus* and *R. rhodnii* depends on the acquisition by first instar nymphs of these bacteria which are present in faeces of the previous generation [37]. In this scenario, plant nutrient sources might add additional elements to the metabolic network of the host-simbiont partnership. This could help to explain the acquisition and maintenance of a more diverse array of microorganisms in the gut of field triatomines.

The present work sets the ground for new questions regarding the eco-epidemiology of Chagas disease. Sugar meals or facultative phytophagy [38, 39] may be a hitherto unobserved feature among triatomines. This behaviour might be an adaptation depending on the association of the insect with plants, or a consequence of extreme poor nutritional status in wild insects. *Rhodnius* spp. and *Panstrongylus megistus* prefer to reside in palms and bromeliads, while some species of *Triatoma* prefer rocky habitats [12, 40]. It is possible that this behaviour relates to plant-nutrient availability.

In brief, we conclusively showed that *R. prolixus* ingests artificial sugar meals and plant material from fruits in the laboratory, impacting its physiology. Further research is necessary to determine to which extent ingested phytochemicals might affect triatomine biology and parasite transmission in nature.

## Conclusions

The triatomine *Rhodnius prolixus* is able to take artificial sugar meals and ingest plant tissue from fruits in the laboratory. Plant nutrient sources improve the fitness of this vector, in terms of life span, blood feeding, mortality post blood feeding and diuresis. These findings challenge the classical view of Triatominae as exclusive blood feeders.
